# Safety and effectiveness of different treatment regimes with tranexamic acid in elective cardiopulmonary bypass patients

**DOI:** 10.1186/cc9850

**Published:** 2011-03-11

**Authors:** JL Iribarren, JJ Jimenez, M Brouard, C Llanos, J Cabrera, L Lorenzo, R Perez, S Palmero, N Perez, L Lorente, M Mora, R Martinez

**Affiliations:** 1 Hospital Universitario de Canarias, La Laguna, Spain

## Introduction

Although tranexamic acid (TA) has been effective in reducing bleeding after cardiac surgery, the TA dosing scheme varies extremely and the agent is highly overdosed in most relevant trials. In a dose-dependent fashion, TA is associated with an increase of adverse events, particularly the observation of seizures. In this study we aimed to assess the safety and effectiveness of different treatment regimes of TA in cardiopulmonary bypass (CPB) patients.

## Methods

A cohort study. The TA treatment regimes were: A: none, B: 40 mg/kg before CPB, C: 25 mg/kg before and 25 mg/kg after CPB, and D: 40 mg/kg before and after CPB. Demographic variables, co-morbidity, perioperative clinical data, and postoperative outcomes (bleeding, RIFLE classification, seizures, stroke and mortality) were recorded. SPSS v15 was used.

## Results

We studied four hundred and five patients (66, 80, 179 and 80 in the A, B, C and D groups, respectively). Surgical procedures were 209 (52%) coronary artery bypass grafting, 135 (33%) valvular, 41 (10%) combined surgery and 20 (5%) other procedures. The 24-hour postoperative bleeding was: A: 992 (95% CI = 808 to 1,177) ml; B: 829 (95% CI = 708 to 950) ml; C: 686 (95% CI = 607 to 765) ml; and D: 671 (95% CI = 550 to 793) ml (F: 18.98, *P *< 0.001). The post-hoc analysis (Scheffé test) showed significant differences between group A versus group C (*P *= 0.002), and between group A versus group D (*P *= 0.003). The 24-hour postoperative red blood cell requirements were A: 384 (95% CI = 248 to 520) ml; B: 200 (95% CI = 119 to 280) ml; C: 253 (95% CI = 184 to 323) ml; and D: 156 (95% CI = 82 to 231) ml (χ^2^: 8.24 *P *= 0.041). We did not find significant differences after surgery between groups regarding the development of stroke (1.3 to 2.5%), RIFLE: I (2.5 to 7.5%) and seizures (0 to 2.5%), even though seizures were present in a dose-dependent fashion.

## Conclusions

A dose higher than 25 mg/kg before and after CPB does not show a clinically relevant decrease in blood loss with a potential increase of adverse events, particularly the observation of seizures.

